# Predictive factors of surgical site infection after hysterectomy for endometrial carcinoma: a retrospective analysis

**DOI:** 10.1186/s12893-021-01264-6

**Published:** 2021-06-14

**Authors:** Lijuan Shi, Qiao Gu, Fenghua Zhang, Daoyun Li, Wenfeng Ye, Yan Zhong, Xiu Shi

**Affiliations:** 1 Department of Obstetrics and Gynecology, The Third Affiliated Hospital of Soochow University, Changzhou, China; 2 Department of Obstetrics and Gynecology, The First Affiliated Hospital of Soochow University, No. 188 Shizi Road, Suzhou, Jiangsu China

**Keywords:** Surgical site infection, Endometrial carcinoma, Prevention, Treatment, Nursing care

## Abstract

**Background:**

Surgical site infection (SSI) is a common postoperative complication. We aimed to analyze the potential risk factors of SSI in patients with endometrial carcinoma.

**Methods:**

Patients with endometrial carcinoma who underwent surgery treatment in our hospital from Sept 1, 2018 to August 31, 2020 were included. We retrospectively compared the characteristics of SSI and no SSI patients, and logistic regression analyses were performed to identify the risk factors of SSI in patients with endometrial carcinoma.

**Results:**

A total of 318 postoperative patients with endometrial carcinoma were included. The incidence of SSI in patients with endometrial carcinoma was 14.47 %. There were significant differences on the FIGO stage, type of surgery, durations of drainage, postoperative serum albumin and postoperative blood sugar (all p < 0.05), and no significant differences on the age, BMI, hypertension, diabetes, hyperlipidemia, estimated blood loss, length of hospital stay were found (all p > 0.05). FIGO stage IV (HR3.405, 95 %CI 2.132–5.625), open surgery (HR2.692, 95 %CI 1.178–3.454), durations of drainage ≥ 7 d (HR2.414,95 %CI 1.125–2.392), postoperative serum albumin < 30 g/L (HR1.912,95 %CI 1.263–2.903), postoperative blood sugar ≥ 10 mmol/L (HR1.774,95 %CI 1.102–2.534) were the independent risk factors of SSI in patients with endometrial carcinoma (all p <  0.05).

**Conclusions:**

Measures including reasonable control of serum albumin and blood glucose levels, minimally invasive surgery as much as possible, timely assessment of drainage and early removal of the tube may be beneficial to reduce the postoperative SSI in in patients with endometrial carcinoma.

## Background

Endometrial carcinoma is a common gynecological malignant tumor, which accounts for 20–30 % of malignant tumors of women’s reproductive systems [1, 2]. Endometrial carcinoma is common in perimenopausal and postmenopausal middle-aged and elderly women [3]. In recent years, the incidence of endometrial carcinoma has been increased and it is the third common gynecological malignant tumor that causes female death [4]. Surgery is the main and effective treatment method for endometrial carcinoma. The combined treatments such as radiotherapy, chemotherapy and endocrine are selected based on the pathological stage and whether there are high-risk factors [5]. With the popularization of medical knowledge and the improvement of health care, more and more patients can timely take endometrial tissue biopsy, gynecological ultrasound, and magnetic resonance imaging (MRI) through segmented diagnostic curettage, hysteroscopy and other clinical methods to early diagnose endometrial carcinoma [6].

Surgery is currently the main method for the treatment of endometrial carcinoma. However, due to the physical characteristics of endometrial carcinoma patients, coupled with the impact of surgery and chemotherapy, the immune system function of patients with endometrial carcinoma is further reduced [7]. It has been reported that the incidence of surgical site infection (SSI) is rather high in patients with endometrial carcinomas [8]. And the incidence of SSI after hysterectomy can be as high as 9–10 % [9, 10]. The infection can affect the healing of the surgical incision, prolong the treatment duration and increase the treatment cost, and the severe infection may have adverse influences on the mortality and prognosis of patients [11, 12]. However, there are few clinical studies on the factors related to SSI after endometrial carcinoma surgery. Therefore, we conducted a retrospective analysis targeted on patients with endometrial carcinoma undergoing surgical treatment, and we analyzed the distribution of pathogenic bacteria and related factors of postoperative SSI, to provide evidence support for the effective prevention and control of SSI in patients with endometrial carcinoma, thereby providing insights into the management of endometrial carcinoma.

## Methods

### Ethical consideration

This study had been checked and approved by the medical ethical committee of The First Affiliated Hospital of Soochow University and The Third Affiliated Hospital of Soochow University, and written informed consents had been obtained from all the included patients.

### Patients

Patients with endometrial carcinoma who underwent surgery treatment in our hospital from Sept 1, 2018 to August 31, 2020 were included. The inclusion criteria of patients were as following: (1) Disease diagnosis met the related diagnostic criteria of endometrial carcinoma [13, 14]. (2) patients took hysterectomy treatment in our department, including laparoscopic and open hysterectomy, the types of surgical selection were based on the patient’s willingness and consents from the doctors after discussing the patient’s condition; (3) patients who did not have serious heart, lung, liver and kidney dysfunction. (4) the patient voluntarily joined this study. Patients with following conditions were excluded: (1) the patients just underwent palliative surgery. (2) patients underwent other operations at the same time. (3) the patient did not agree to participant in our study.

### Hysterectomy treatment

We perform surgery on the patient under general anesthesia. The patient was in a state of head down and feet high and kept tilted at 30°. During the operation, the electrocardiogram, airway pressure and blood oxygen were monitored, and urinary catheters and other utensils were all placed. Open hysterectomy was performed as usual. For laparoscopic hysterectomy, we took the patient’s umbilicus with the pressure of the pneumoperitoneum of 12–13 mmHg to insert the first 10 nllTl Trocar, and the operation used a 4-point approach. The cutting tools were ultrasonic knife and scissors, and we used monopolar and bipolar electrocoagulation to stop bleeding and suture accordingly.

### Diagnostic criteria

The diagnosis, classification and staging criteria of endometrial carcinoma were conducted in accordance with the “Guidelines for the Diagnosis and Treatment of Endometrial Carcinoma“ [15] based on the patient’s medical history, clinical manifestations, general examinations and gynecological examinations, uterine tissue biopsy. Postoperative pathological tissue biopsy was the gold standard for diagnosis of endometrial carcinoma. International Federation of Obstetrics and Gynecology (FIGO) staging was based on histological grade, nuclear grade, depth of invasion, site of invasion, metastasis, ascites cytology [14].

The diagnostic criteria for SSI was based on the “Technical Guidelines for Surgical Site Infection Prevention and Control” and “Diagnosis of Nosocomial Infections” promulgated by the Chinese National Health Commission [16], that was, surgery-related infections of subcutaneous tissue, incision skin, fascia and muscle layer that occurred within 30 days after surgery. Our hospital strictly controlled the antibiotics use, and all the included patients did not accept the pre-operative antibiotic prophylaxis. But after the operation, we routinely used latamoxef 2 g/day for three days to prevent infections.

### Data collection

Two authors independently collected following data: patient’s age, body mass index (BMI), hypertension, diabetes, hyperlipidemia, FIGO stage, results of bacteria test, type of surgery, estimated blood loss, durations of drainage, length of hospital stay, postoperative serum albumin and postoperative blood sugar level.

### Statistical analysis

After double checking the collected data, all the data were input into SPSS 23.0 statistical software for data analysis. We conducted t test and Chi square tests to analyze the characteristics and treatment details of patients. Besides, logistic regression analyses were conducted to identify the potentially influencing factors for SSI in patients with endometrial carcinoma after surgery treatment. p < 0.05 was considered statistically significant in this study, and all the tests were two-sided.

## Results

### Patient inclusion

A total of 318 postoperative patients with endometrial carcinoma were included. The characteristics of included 318 patients with endometrial carcinoma were presented in Table 1. There were significant differences on the FIGO stage, type of surgery, durations of drainage, postoperative serum albumin and postoperative blood sugar between two groups(all p < 0.05), the patients with FIGO stage IV, open surgery, durations of drainage ≥ 7d, postoperative serum albumin < 30 g/L and postoperative blood sugar ≥ 10 mmol/L in SSI group were significant more than that of no-SSI group, indicating that e FIGO stage, type of surgery, durations of drainage, postoperative serum albumin and postoperative blood sugar might be associated with the onset and development of SSI in patients with endometrial carcinoma. No significant differences on the age, BMI, hypertension, diabetes, hyperlipidemia, estimated blood loss, length of hospital stay between two groups were found (all p > 0.05), indicating that those factors might not be associated with SSI in patients with endometrial carcinoma.

**Table 1:** The characteristics of included patients with endometrial cancer

Variables	SSI patients (n = 46)	No SSI patients (n = 272)	χ^2^	p
Age (y)				
≥ 60	36 (78.26 %)	210 (77.21 %)	1.024	0.089
< 60	10 (21.73 %)	62 (22.79 %)		
BMI(kg/m^2^)				
≥ 25	15 (32.61 %)	90 (33.09 %)	1.105	0.121
< 25	31 (67.39 %)	182 (66.91 %)		
Hypertension				
Yes	34 (73.91 %)	201 (%)	1.217	0.104
No	12 (26.09 %)	71 (26.10 %)		
Diabetes				
Yes	19 (41.30 %)	109 (40.07 %)	1.095	0.112
No	27 (58.70 %)	163 (59.93 %)		
Hyperlipidemia				
Yes	11 (23.91 %)	61 (22.43 %)	1.022	0.104
No	35 (76.09 %)	211 (77.57 %)		
FIGO staging				
Stage I	6 (13.04 %)	95 (34.93 %)	1.187	0.018
Stage II	15 (32.61 %)	73 (26.84 %)		
Stage III	13 (28.26 %)	57 (20.96 %)		
Stage IV	12 (26.09 %)	47 (17.28 %)		
ASA staging				
Stage I	14 (30.43 %)	88 (32.35 %)	1.204	0.096
Stage II	13 (28.26 %)	81 (29.78 %)		
Stage III	9 (19.57 %)	53 (19.49 %)		
Stage IV	10 (21.74 %)	50 (18.38 %)		
Stage V	0 (0 %)	0 (0 %)		
Type of surgery				
Laparoscopic surgery	18 (39.13 %)	168 (61.76 %)	1.185	0.022
Open surgery	28 (60.87 %)	104 (38.23 %)		
Estimated blood loss(ml)				
< 100	19 (41.31 %)	117 (43.01 %)	1.123	0.123
≥ 100	27 (58.70 %)	150 (55.15 %)		
Durations of drainage(d)				
< 7	21 (45.65 %)	142 (52.21 %)	1.236	0.031
≥ 7	25 (54.35 %)	130 (47.79 %)		
Length of hospital stay(d)				
≥ 14	10 (21.74 %)	41 (15.07 %)	1.131	0.063
< 14	36 (78.26 %)	231 (84.93 %)		
Postoperative serum albumin(g/L)				
≥ 30	16 (34.78 %)	139 (51.11 %)	1.120	0.043
< 30	30 (65.22 %)	133 (48.89 %)		
Postoperative blood sugar (mmol/L)				
≥ 10	33 (71.73 %)	94 (34.56 %)	1.317	0.011
< 10	13 (28.26 %)	178 (65.44 %)		

### SSI pathogen distribution

Of the included 318 patients, 46 patients had SSI, the incidence of SSI in patients with endometrial carcinoma was 14.47 %. The Gram-negative bacteria was the most commonly-seen pathogen of SSI (71.74 %). The pathogen distribution of SSI in patients with endometrial carcinoma was showed in Table 2.

**Table 2:** The pathogen distribution of SSI in patients with endometrial cancer

Pathogen	Cases	Percent (%)
Gram-positive bacteria	12	26.09
*Staphylococcus aureus*	6	13.04
*Staphylococcus epidermidis*	3	6.52
*Enterococcus faecalis*	2	4.35
*Streptococcus pneumoniae*	1	2.17
Gram-negative bacteria	33	71.74
*Escherichia Coli*	15	32.61
*Klebsiella pneumoniae*	8	17.39
*Pseudomonas aeruginosa*	5	10.87
*Enterobacter cloacae*	2	4.35
*Acinetobacter baumannii*	2	4.35
*Enterobacter aerogenes*	1	2.17
Fungus	1	2.17
*Candida albicans*	1	2.17

### The risk factors for postoperative SSI in patients with endometrial carcinoma

We conducted logistic regression analyses for the risk factors for SSI in patients with endometrial carcinoma. As showed in Table 3, FIGO stage IV (HR3.405, 95 %CI 2.132–5.625), open surgery (HR 2.692, 95 %CI 1.178–3.454), durations of drainage ≥ 7 d (HR2.414,95 %CI 1.125–2.392), postoperative serum albumin < 30 g/L(HR1.912,95 %CI 1.263–2.903), postoperative blood sugar ≥ 10 mmol/L (HR1.774,95 %CI 1.102–2.534) were the independent risk factors for SSI in patients with endometrial carcinoma (all p < 0.05).

**Table 3:** Logistic regression analysis on the risk factors for SSI in patients with endometrial cancer

Variables	HR	95 %CI	p
FIGO Stage IV	3.405	2.132–5.625	0.003
Open surgery	2.692	1.178–3.454	0.011
Durations of drainage ≥ 7 d	2.414	1.125–2.392	0.014
Postoperative serum albumin < 30 g/L	1.912	1.263–2.903	0.034
Postoperative blood sugar ≥ 10 mmol/L	1.774	1.102–2.534	0.027

## Discussions

Endometrial carcinoma is a common carcinoma among gynecological malignancies. At present, endometrial carcinoma has become the first gynecological malignant tumor in some parts of China, and the patients with endometrial carcinoma is becoming younger and younger [17, 18]. Most endometrial carcinoma patients have early clinical symptoms, such as irregular vaginal bleeding after menopause, increased or prolonged menstruation, abnormal vaginal discharge and so on [5, 19]. At present, surgery based on FIGO staging is still the main treatment for patients with endometrial carcinoma [20]. SSIs are infections of incisions or deep organs or cavities during the perioperative period [21]. It is one of the common types of nosocomial infections [22]. The results of our study have found that the incidence of SSI in patients with endometrial carcinoma is 14.47 %, and patients with FIGO stage IV, open surgery, durations of drainage ≥ 7d, postoperative serum albumin < 30 g/L, postoperative blood sugar ≥ 10 mmol/L may have higher risks for SSI, early preventive measures and treatments are needed for those patients.

The incidence of SSI in patients with endometrial carcinoma in this present study is higher than the results of SSI of patients with non-malignant tumor operation in previous studies [23, 24], indicating that patients with endometrial carcinomas have higher risk of SSI, which may be related to that malignant tumors can disrupt the patient’s immune system and reduce anti-infection ability [25, 26]. Besides, 46 strains of pathogenic bacteria were isolated from patients with SSI, which are mainly gram-negative bacteria, among which Escherichia coli, Klebsiella pneumoniae and Staphylococcus aureus were more common. The findings of the distribution of SSI pathogenic bacteria in previous studies [27, 28] are consistent with our results. There is no significant difference in the distribution of pathogens at the surgical site of endometrial carcinoma and other gynecological surgical sites, which may be associated with the same characteristics of colonizing pathogens at the same surgical site [29]. The pathogen distribution of SSI in patients with endometrial carcinoma can provide a reference for the clinical selection of antibiotics [30].

There are many factors that may be related to the onset and development of SSI. The elderly patients have many basic diseases, the body’s immune function is low, and the risks of SSI increase accordingly [31]. The FIGO staging include the comprehensive histological grade, nuclear grade, depth of invasion, site of invasion, metastasis, ascites cytology, which are used to guide the development of treatment plans and prognosis for endometrial carcinoma. Most patients with stage III and IV have metastasized and need to expand the scope of surgery [32]. And the tumor is difficult to completely remove, not only the prognosis of treatment is poor, but also the incidence of SSI is higher [33]. For the patients with diabetes, especially those with poor blood sugar control, have reduced body defenses and reduced ability to resist the invasion of foreign pathogenic microorganisms, which can increase risks of SSI [30]. Previous studies [27, 28] have reported that patients with higher BMI ​​have thicker subcutaneous fat tissue, poor blood flow to adipose tissue, and are prone to fat liquefaction, with are associated with the development of SSI. We have not found the relationship between BMI and SSI, which may be related to the fact that the sample size is small, future studies with larger sample size are needed.

Laparoscopy and other minimally invasive surgical methods have several advantages including small incisions, less intraoperative blood loss, reduced tissue trauma, and shortened surgical exposure time, which can reduce the incidence of SSI [34, 35]. Postoperative drainage of the surgical site can drain the deep exudate, which is conducive to surgical healing, but the longer duration of drainage can increase the exposure of the surgical site, and the drainage may bring pathogenic bacteria into the surgical site, thereby increasing the risks of SSI [36]. Endometrial carcinoma is one of the common malignant tumors in gynecology, the catabolism of albumin increases accordingly, the amount of albumin required for repairing surgical site increases [37]. Lower albumin level indicates the lack of albumin, which is harmful to the recovery of surgical site [38, 39]. The intimate detection of serum albumin level is necessary for the postoperative patients. Previous studies [40, 41] have analyzed the risk factors of early and late postoperative complications in patients with endometrial carcinoma. It has been found that higher blood sugar level is an independent risk factor for early postoperative complications, which is consistent with our findings. Therefore, the appropriate control and monitor of blood sugar is vital to the management of SSI [42].

Several limitations in this present study must be concerned. Firstly, we selected patients with endometrial carcinoma who underwent surgery treatment in our hospital from Sept 1, 2018 to August 31, 2020 as the study population, and we tried to collect related data as much as possible, since our study was a retrospective analysis, many data could not be included for analysis. Secondly, we did not calculate the sample size, the sample size might underpower to detect the potential risks. Thirdly, postoperative complications based on Clavien Dindo [43] should be included for analysis, limited by data, we could not include the related complications for further analysis. Furthermore, we did not include the organ deep specific infection and postoperative intra-abdominal collection for consideration, those serious infection cases should also be concerned. Even through there are many previous studies on this topic, but our study does have same strengths on the results of postoperative serum albumin < 30 g/L, postoperative blood sugar ≥ 10 mmol/L, which may provide specific guidance to the clinical management of postoperative serum albumin and blood sugar management. Besides, we think that the similar topic should be reported in different region and population. Future studies with prospective design and larger-sample size are warranted to further elucidate the potential risks of SSI in patients with endometrial carcinoma, to provide reliable evidence to the management of endometrial carcinoma.

## Conclusions

In summary, SSI is common after surgery in patients with endometrial carcinoma. Effective reduction and control of SSI is essential to the success of surgery treatment. Therefore, it is particularly important to actively prevent SSI. Minimally invasive surgery should be used as much as possible with improved surgical skills, and health care providers should reduce unnecessary drainage of the surgical site, timely evaluate and remove the drainage tube early after the operation, and reasonably control the patient’s serum albumin and blood sugar levels to improve the prognosis of patient.

## Data Availability

We would like to share the raw data for the scholars who are interested in our study, please contact Xiu Shi by email shixiudrdr@21cn.com in that case.

## Abbreviations

SSI: Surgical site infection
MRI: Magnetic resonance imaging
FIGO: International Federation of Obstetrics and Gynecology
BMI: Body mass index
